# Short communication: The miR-155a-5p is correlated with increased ROS and impaired apoptosis in macrophages infected by *Leishmania braziliensis*

**DOI:** 10.1371/journal.pone.0298458

**Published:** 2024-02-21

**Authors:** Tainã Lago, Thyago Marconi Cardoso, Alan Rocha, Edgar M. Carvalho, Léa Cristina Castellucci

**Affiliations:** 1 Serviço de Imunologia da Universidade Federal da Bahia, Salvador, Brazil; 2 Programa de Pós-graduação em Ciências da Saúde da Universidade Federal da Bahia, Salvador, Brazil; 3 Instituto Nacional de Ciência e Tecnologia em Doenças Tropicais, Salvador, Brazil; 4 Laboratório de Pesquisas Clínicas—LAPEC—Instituto Gonçalo Moniz-FIOCRUZ, Salvador, Bahia, Brazil; Kerman University of Medical Sciences, ISLAMIC REPUBLIC OF IRAN

## Abstract

Cutaneous leishmaniasis (CL) caused by *Leishmania braziliensis*, is a disease characterized by well-limited ulcerated lesions with raised borders in exposed parts of the body. miRNAs are recognized for their role in the complex and plastic interaction between host and pathogens, either as part of the host’s strategy to neutralize infection or as a molecular mechanism employed by the pathogen to modulate host inflammatory pathways to remain undetected. The mir155 targets a broad range of inflammatory mediators, following toll-like receptors (TLRs) signaling. In this work, we evaluated the effects of the expression of miR155a-5p in human macrophages infected with L. braziliensis. Our results show that miR155a-5p is inversely correlated with early apoptosis and conversely, seems to influence an increment in the oxidative burst in these cells. Altogether, we spotted a functional role of the miR155a-5p in CL pathogenesis, raising the hypothesis that an increased miR-155 expression by TLR ligands influences cellular mechanisms settled to promote both killing and control of parasite density after infection.

## Introduction

Cutaneous leishmaniasis (CL) caused by *Leishmania braziliensis* infection is characterized by an exaggerated inflammatory response that controls the parasite burden but also contributes to pathology [1]. The disease is characterized by one or more ulcers with raised borders, usually located on uncovered parts of the body. Ulcers may eventually self-heal but often take months, leading to disfiguring and treatment-resistant lesions, whose refractoriness to pentavalent antimony (is a first choice drug for decades) can reach up to 50% [2–4]. Moreover, 3% of subjects infected with *L*. *braziliensis* will develop the mucosal disease in addition to emerging forms such as disseminated as well as atypical leishmaniasis [5–8]. MicroRNAs (miRNAs), a subset of non-coding RNAs are ∼22-nt long endogenously initiated short RNA molecules post-transcriptionally regulating cleavage of target mRNAs or just repressing their translation [9]. The miR-155, triggered after the TLRs signaling contributes to different pathways such as apoptosis, inflammation and immunity, in which apoptosis induction is by targeting caspase-3 mRNA [10]. Moreover, miR155 has also been implicated in the production of reactive oxygen species (ROS) in different models [11–13]. Considering the importance of this miRNA in the immune response, we investigated if the miR-155a is associated with CL, infecting macrophages with an *L*. *braziliensis* isolate from a CL patient and analyzing the production of markers of cell death expression (apoptosis, necrosis) and oxidative burst.

## Material and methods

### Culture of *L*. *braziliensis* isolates

An isolate of *L*. *braziliensis* (BR/30035) was obtained from a skin lesion of a patient with CL derived from the endemic area of Corte de Pedra, Bahia, Brazil. The parasites were initially cultured in biphasic medium Novy-MacNeal-Nicolle (NNN) (modified blood agar) and LIT (Liver Infusion Tryptose). Following isolation, the parasites were cryopreserved in frozen nitrogen. Prior use, the isolate was expanded in Schneider medium supplemented with 10% fetal bovine serum (FCS) and 2% urine and cultivated in a incubator until 24°C. For seven days, the growth cycle of parasites was monitored by counting viable promastigotes in order to assess the different stages of *L*. *braziliensis* growth until the stationary phase.

### Macrophage acquisition and infection

Peripheral blood mononuclear cells (PBMCs) from ten healthy volunteers, both sexes, aged between 25–50 years, were collected between 03^th^ July 2019 to 28^th^ January 2020. These individuals are immunocompetent and are not resident leishmaniasis endemic area, S1 Table. Cells were obtained from heparinized blood using Ficoll Hypaque™ Plus density Gradient (GE healthcare, Biosciences AB Durham, NC, USA) and incubated in Teflon flasks for 6 days at 37°C and 5% CO2 to differentiate monocytes into macrophages. After this incubation period, the cells were washed, adjusted to 1x10^6^ and cultured in 12-well culture plates for 24 h for macrophage adherence. Afterwars, macrophage cultures (1x10^6^ cells/well) were infected with *L*. *braziliensis* promastigotes in the stationary phase at a ratio of 5:1 and uninfected macrophages were used as controls. The infection rate was monitored by optical microscopy counting the percentage of infected macrophages and the number of amastigotes per 200/cells in duplicate, at times of 4, 12 and 24 h. We used the kit Panótico Rápido (Laborclin, Brasil) a rapid staining set in hematology that contains dyes with chemical characteristics and affinities, according to the manufacturer’s instructions.

### RNA extraction, cDNA sytensis and evaluation of miRNA expression by quantitative RT-PCR (qPCR)

The cells RNA was extracted using the TRIzol® Reagent method, according to manufacturer’s instructions. The isolated RNA was resuspended in 25μl of RNAse-free water and concentration was determined by optical density measurements (260 and 280 nm) using Nanodrop®. Complementary DNA (cDNA) was obtained using the miRCURY LNA™ RT Kit (QIAGEN), following the manufacturer’s guidelines. The expression of the miR155a as well as the normalizers 146b-5p and U6snComplementary DNA (cDNA) were performed using qPCR, through the miRCURY LNA SYBR Kit. Briefly: 5μl of 2x miRCURY LNA SYBR Green Master mix, 0.05μl of ROX Reference Dye, 1μl of PCR primer mix and 1μl of RNase-free water. A final product of 10μl was used for each reaction containing 3μl of the diluted cDNA template (1:5) and 7μl of the reaction mix. The amplification conditions followed the following cycling (I) 2 minutes at 95°C, (II) 10 seconds at 95°C, (III) 60 seconds at 56°C (IV) for 40 cycles following the melting curve. After normalization, gene expression unites were calculated from values of ΔCt.

### Evaluation of cell death and oxidative burst

Flow cytometry was used to access apoptotic/necrotic cells and determine NOS and ROS species expression. After 48 h, cultured cells were labeled with 5 μL PE–anti-CD14 MAb (clone 61D3; BD-Bioscience Pharmingen, San Jose, CA). Afterward, 5 μL of the annexin V (AnV) FITC in the annexin binding buffer (BD-Bioscience Pharmingen) was added for 20 min. Samples were analyzed on a FACS Fortessa flow cytometer (BD-Bioscience Pharmingen, San Jose, CA). To access NOS and ROS intracellular species cells were labelled with dihydrorhodamine 123, DAF-FM diacetate (Life Technologies), dihydroethidium (Life Technologies) and CM-H2DCFDA (Invitrogen—Thermo Fisher Scientific), diluted according to the manufacturer’s instructions. Viable cells (AnV-/IP-) after 48h were counted. A minimum of 50,000 gated events from each sample were collected in a FACS Fortessa flow cytometer (BD-Bioscience Pharmingen, San Jose, CA) and analyzed using the FlowJo 7.6.5 program.

### Data analysis

The gene expression was normalized using the RefFinder program. We used the Delta-Ct comparative method to represent relative expression units between CL and unfected macrophages within each time point. After normalization, comparisons were made using the Mann-Whitney test. Correlation analysis with AnV and ROS was performed using the Spearman correlation test. Statistics were implemented by the GraphPad Prism 8 software. P values < 0.05 were considered statistically significant.

### Ethics statement

The study was approved by the Research Ethics Committee of the Complexo Hospitalar Professor Edgard Santos of the Federal University of Bahia (Opinion 3,020,979) in compliance with the recommendation of Resolution 466/12 of the National Health Council for research on human beings and the Declaration of Helsinki. Participants signed informed consent prior sample collection.

## Results

**Fig 1:**
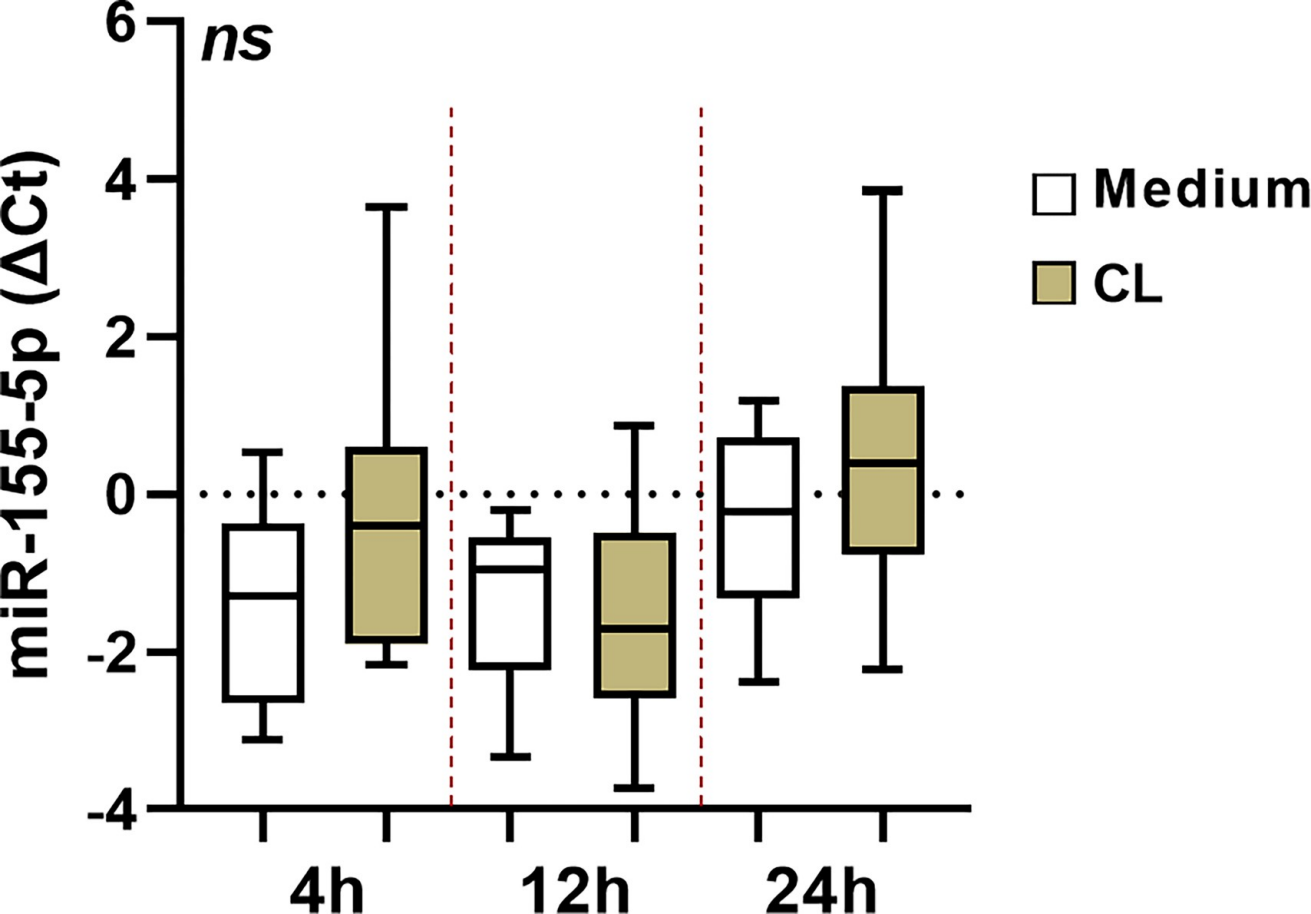
Difference in miR-155-5p expression in *L*. *braziliensis* infected cells and uninfected cells. Expression of miRNAs in macrophages infected with the isolate BR/30035 of *L*. *braziliensis* at different times of infection (4h, 12h and 24h). Data is represented in log fold change.

**Fig 2:**
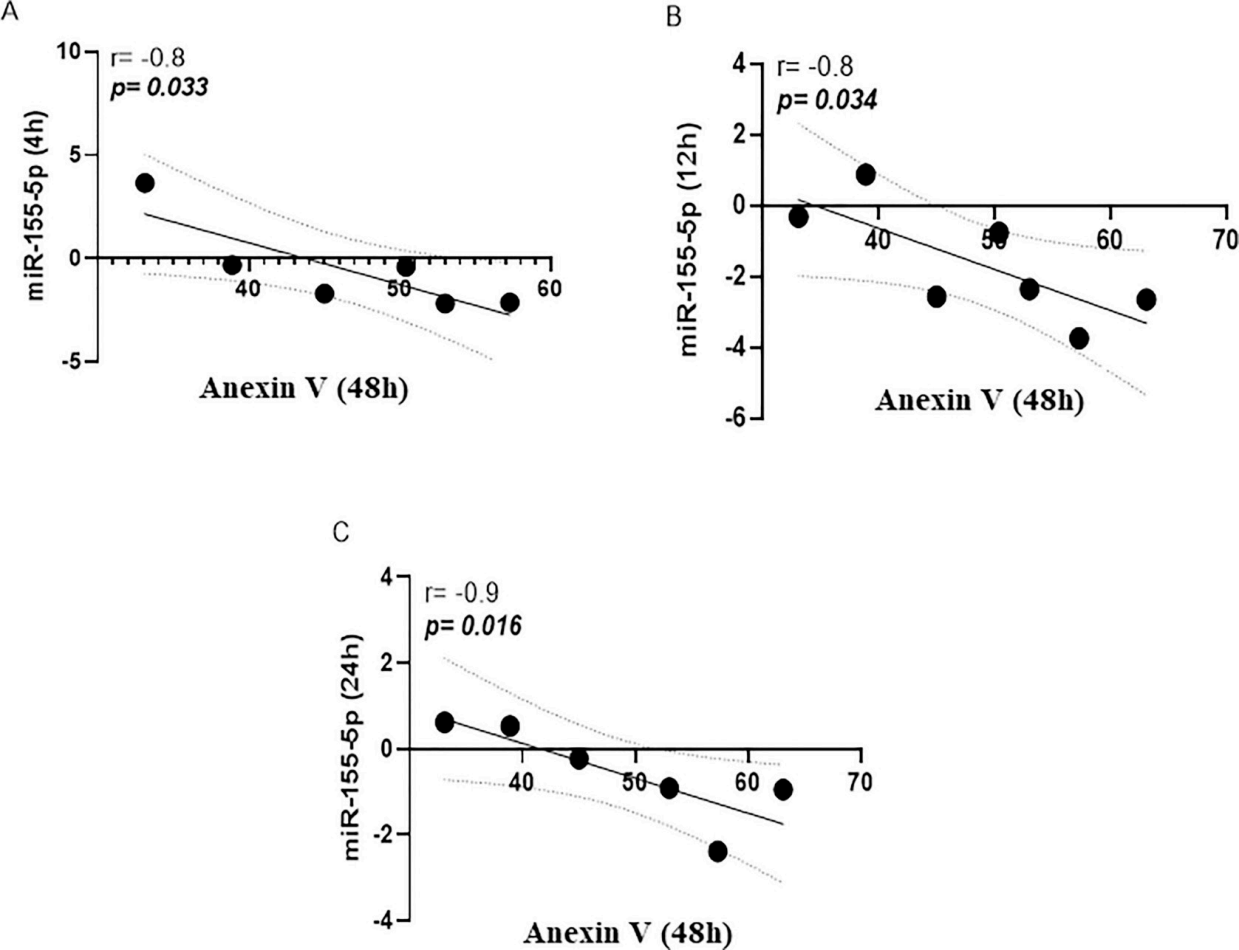
Inverse correlation between the miR-155a-5p expression and apoptosis. At three time points tested there was a consistent negative correlation between cells annexin positive (Anv+) and the miR expression: 4h (r = -0.8, p = 0.033), 12h (r = -0.8, p = 0.034) and 24h (r = -0.9, p = 0.016), A, B and C, respectively, in macrophages infected with *L*. *braziliensis* (BR/30035). Analysis made by Spearman correlation test, implemented by the GraphPad Prism program V.8.0.

**Fig 3:**
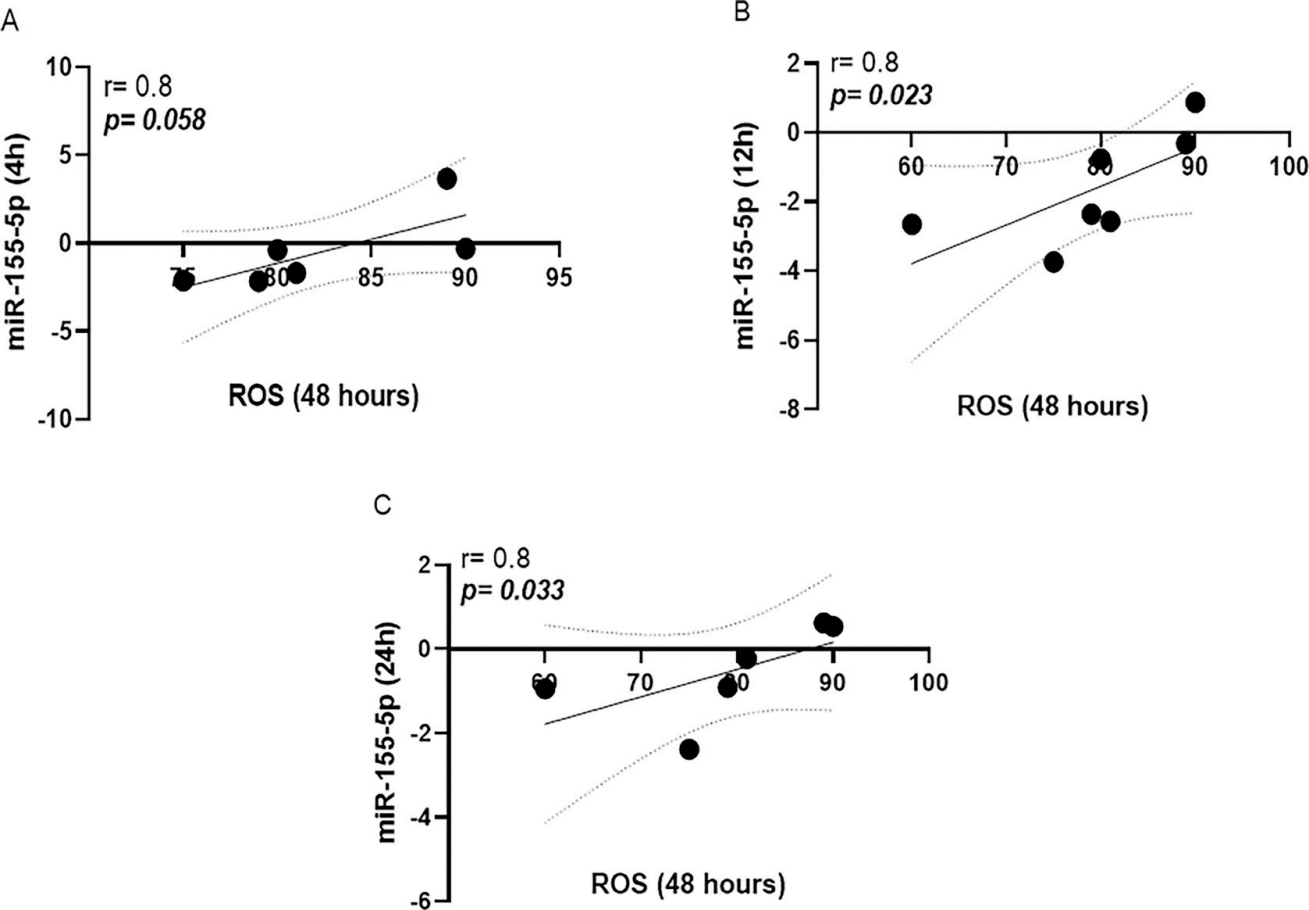
Positive correlation between oxidative burst and the miR-155a-5p expression. There was a positive correlation between cells expressing ROS (DHR 123) at 4h (r = 0.8, p = 0.058), 12h (r = 0.8, p = 0.023) and 24h (r = 0.8, p = 0.033), A, B and C, respectively in macrophages infected with *L*. *braziliensis* (BR/30035). Analysis made by Spearman correlation test, implemented by the GraphPad Prism program V.8.0.

**Fig 4:**
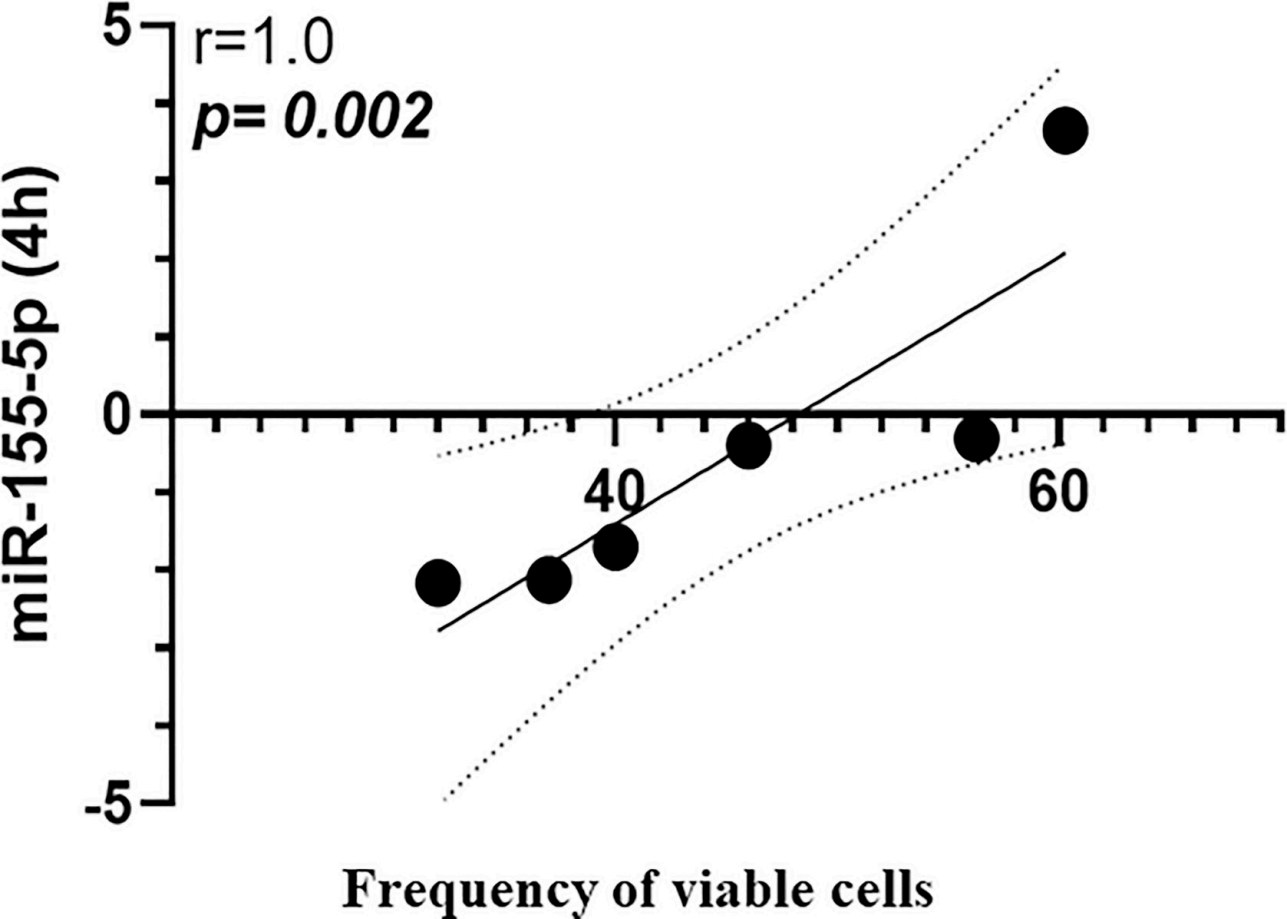
Mir-155a-5p correlates with viable cells in culture. In the first h post-infection, the miR-155a-5p expression correlates positively with the number of viable macrophages in culture (non-apoptotic cells) for up to 48h in culture. Analysis made by Spearman correlation test, implemented by the GraphPad Prism program V.8.0.

While there were no significant differences in miR-155a-5p expression in uninfected versus *L*. *braziliensis* infected cells, Fig 1. A strong inverse correlation between the frequency of Annexin expressing cells (Anv+) and miR-155a-5p expression was documented at all three times points: 4h (r = -0.8, p = 0.033), 12h (r = -0.8, p = 0.034) and 24h (r = -0.9, p = 0.016), respectively, as shown in Fig 2. Conversely, a positive correlation was observed between the oxidative burst and the expression of this miRNA at 4h (r = 0.8, p = 0.058), 12h (r = 0.8, p = 0.023) and 24h (r = 0.8, p = 0.033), Fig 3. Additionally, the early expression of this miRNA after infection was positively correlated with the number of viable cells in culture (non-apoptotic cells) for up to 48 h, Fig 4.

## Discussion

In *L*. *braziliensis* infection while the oxidative burst results in parasite killing, apoptosis is associated with pathology. The activation of cell death has been correlated with tissue damage in CL [14, 15]. Consistent with this, targets of granzyme B include activation of caspase-3 directly or through the mitochondrial pathway, by inducing activation of caspase-9, which in turn activates caspase-3, amplifying the caspase cascade [16]. Additionally, a proteomic analysis comparing lesion *versus* normal skin showed a positive correlation between CL and expression of caspase-9, caspase-3 and granzyme, data that corroborate cell death mechanisms with the local tissue destruction due infection by *L*. *braziliensis* [17]. Alternatively inhibition of miR-155 increased the number of apoptotic cells and caspase-3/7 expression, whereas the parasite load decreased in the spleen of the mice infected with *L*. *major* [18]. Data have pointed out the role of miR-155 inhibiting apoptosis likely by targeting Caspase-3 and Caspase-9 mRNA, and the PTEN signaling pathways [19–21]. Based in our results, we raise the hypothesis that miR-155 may act to promote anti- apoptotic effects, and this acts in favor of controlling lesion progress after the establishment of the infection by *L*. *braziliensis*. This is consistent with the correlation we observed between this miRNA and an increased number of viable cells after 48h.

The miR-155 was positively correlated with ROS production which is one of the main mechanisms used by macrophages from *Leishmania* killing. It is recognized that patients with active CL produce high levels of INF- γ and TNF-α, cytokines that contribute to the production of ROS, favoring parasite control. However, despite macrophages produce ROS and NO, sufficient *Leishmania* persists in host cells leading to disease progress [22, 23]. In addition, in monocytes of CL patients infected by *L*. *braziliensis* an inhibition of ROS was detected which allowed the growth of viable promastigotes in culture supernatants. On the other hand, a positive correlation between NO production and lesion size was also observed, suggesting that while the production of ROS is involved in *L*. *braziliensis* killing, NO alone is not sufficient to contain infection and may add to the tissue damage observed in human CL [24]. Additionally, data have shown that use of miR-155 inhibitor in cell cultures increased the apoptosis of infected macrophages in vitro, and was also correlated with a reduction in the lesions size at six weeks in vivo [18]. This data is put in a perspective that miRNA-based therapy could be a possible toll to treat cutaneous leishmaniasis.

Overall, we suggest a role for the miR-155 in the regulation of both, apoptosis and ROS production that might be particularly important for parasite killing after *L*.*braziliensis* enter host macrophages. Nevertheless, more sophisticated cell assays with mimics and inhibitors will be important to confirm these findings as well as to test other possible impacts on the host´s immune response.

## Supporting information

S1 Table Demographic data (Age and sex) of study participants. Values provided as average with standard deviation (SD) and frequency, respectively.
(DOCX)
