# Supplementary material for: Short communication: The miR-155a-5p is correlated with increased ROS and impaired apoptosis in macrophages infected by Leishmania braziliensis
Source: PLoS One. 2024 Feb 21;19(2):e0298458. doi: 10.1371/journal.pone.0298458 (PMC10880991; doi:10.1371/journal.pone.0298458)
Supplement: S1 Table — Values provided as average with standard deviation (SD) and frequency, respectively. (DOCX) [file pone.0298458.s001.docx]

Table 1. Demographic data (Age and sex) of study participants.

|  | **Healthy volunteers (N=10)** |
| --- | --- |
| **Age, mean (SD), years** | 30 ± 7 |
| **Sex** |  |
| **Male, n/N (%)** | 6/10 (60%) |
| **Female, n/N (%)** | 4/10 (40%) |
| Values provided as average with standard deviation (SD) and frequency, respectively. | |
